# An Analysis of the Areas Occupied by Vessels in the Ocular Surface of Diabetic Patients: An Application of a Nonparametric Tilted Additive Model

**DOI:** 10.3390/ijerph18073735

**Published:** 2021-04-02

**Authors:** Farzaneh Boroumand, Mohammad Taghi Shakeri, Touka Banaee, Hamidreza Pourreza, Hassan Doosti

**Affiliations:** 1 Department of Mathematics and Statistics, Faculty of Science and Engineering, Macquarie University, Sydney 2109, Australia; Farzaneh.Boroumand@mq.edu.au; 2 Department of Biostatistics, School of Health, Mashhad University of Medical Sciences, Mashhad 9137673119, Iran; 3 Department of Ophthalmology and Visual Sciences, University of Texas Medical Branch, Galveston, TX 77555, USA; Tobanaee@utmb.edu; 4 Department Computer Engineering, Faculty of Engineering, Ferdowsi University of Mashhad, Mashhad 9177948974, Iran; Hpourreza@um.ac.ir

**Keywords:** diabetes, ocular surface, area occupied by vessels, metabolic syndrome, generalized additive model, nonparametric regression, tilted estimator, bootstrap confidence band

## Abstract

(1) Background: As diabetes melllitus (DM) can affect the microvasculature, this study evaluates different clinical parameters and the vascular density of ocular surface microvasculature in diabetic patients. (2) Methods: In this cross-sectional study, red-free conjunctival photographs of diabetic individuals aged 30–60 were taken under defined conditions and analyzed using a Radon transform-based algorithm for vascular segmentation. The Areas Occupied by Vessels (AOV) images of different diameters were calculated. To establish the sum of AOV of different sized vessels. We adopt a novel approach to investigate the association between clinical characteristics as the predictors and AOV as the outcome, that is Tilted Additive Model (TAM). We use a tilted nonparametric regression estimator to estimate the nonlinear effect of predictors on the outcome in the additive setting for the first time. (3) Results: The results show Age (*p*-value = 0.019) and Mean Arterial Pressure (MAP) have a significant linear effect on AOV (*p*-value = 0.034). We also find a nonlinear association between Body Mass Index (BMI), daily Urinary Protein Excretion (UPE), Hemoglobin A1C, and Blood Urea Nitrogen (BUN) with AOV. (4) Conclusions: As many predictors do not have a linear relationship with the outcome, we conclude that the TAM will help better elucidate the effect of the different predictors. The highest level of AOV can be seen at Hemoglobin A1C of 9% and AOV increases when the daily UPE exceeds 600 mg. These effects need to be considered in future studies of ocular surface vessels of diabetic patients.

## 1. Introduction

Diabetes mellitus (DM) is a metabolic disease defined according to an individual’s level of hyperglycemia [1]. Recent estimates show that, globally, 463 million (9.3%) people live with DM. It is projected that this figure will increase to 578 million (10.2%) in 2030, and 700 million (10.9%) in 2045 [2]. The increasing prevalence of DM is concerning as it can lead to more people experiencing the complications of diabetes. Diabetes-related complications can cause damage to the body’s organs in three ways. First, they can create macrovascular or cardiovascular complications that lead to heart attack, stroke, or circulation problems in the lower limbs [3]. Second, diabetes can create microvascular complications causing problems in the eyes, kidneys, feet, and nerves. The most prominent microvascular complications are retinopathy, nephropathy, and neuropathy [3]. Third, they can affect other parts of the body including, skin (diabetic dermopathy) [4], teeth, and gums as a result of increased risk of infection [5].

In this study, we focus on microvascular complications in the ocular surface. The ocular surface refers to the conjunctiva and corneal epithelium, which protect the eye against microbes, trauma, and toxins [6]. DM can affect the ocular surface by increasing the risk of developing a dry eye disease [7], corneal epithelial fragility, decreased corneal sensitivity, and abnormal wound healing [8]. Diabetic retinopathy is one of the most important microvascular complications of diabetes. Up to now, most studies documenting the microvascular changes of diabetes have focused on the retina, but photography of the retina requires expensive fundus cameras and expert photographers, which may not be available [9,10,11]. For the purpose of this study, we analyze the vessels visible over the surface of the eye, which include both conjunctival and episcleral vessels. Hence, we explore whether the microvasculature of the ocular surface, which is easier to see and photograph, is affected by diabetes or otherwise. In a previous paper [12], we showed that the microvasculature of the ocular surface is affected by development of diabetes. That study used a regression model, which did not show any effect of different predictors on the ocular surface vessels. The current study differs in using a novel statistical method to find possible nonlinear effects of the predictors. However, studying conjunctival vessels is difficult since they rapidly react to irritations. Recently, improvements in digital photography and image processing mean that the examination of conjunctival vessels is more achievable. As a result, there is growing interest in the study of conjunctival vessels [11,13,14,15,16].

In the current study, we analyse a relatively large number of ocular surface images from diabetic patients to assess the association between demographic and clinical characteristics in relation to the Areas Occupied by Vessels (AOV) in the ocular surface. To investigate the association between clinical characteristics as the predictors and AOV as the outcome, we need to find an appropriate statistical method. The Generalized Linear Model (GLM) is a powerful methodology used to identify associations in medical research [17].

In GLM, we fit the following model

(1) $$g\left(E\left(Y\right)\right)=\alpha +\beta_{1}X_{1}+\beta_{2}X_{2}+\;\ldots \;+\beta_{p}X_{p},$$

where *Y* is an outcome, $X_{1},\;\ldots \;,X_{p}$ are the predictors, $\beta_{i}$ are unknown regression coefficient parameters, and g(.) is a link function [18]. It is worth noting that this model is linear with respect to the coefficients, not the predictors. Linear regression is a special case of GLM while the link function is an identity function

(2) $$E\left(Y\right)=\alpha +\beta_{1}X_{1}+\beta_{2}X_{2}+\;\ldots \;+\beta_{p}X_{p}.$$

With fitting model (2), we assume that a line with slope $\beta_{i}$ reflects the association between the outcome and the $i^{th}$ predictor, $X_{i}$. In fact, in testing this hypothesis $\beta_{i}=0$, it is important to note that a linear relationship will be tested, as this relationship might be nonlinear. The GLM cannot detect the nonlinear association (nonlinearity in terms of coefficients); instead, this issue can be addressed using a parametric transformation. However, parametric transformation may not fit the data well. An alternative solution might be to use a nonparametric method as a data-driven approach to let the data describe the form of the association itself [19]. In a nonparametric approach, GLM can be replaced by Generalized Additive Model (GAM) for adjusting, predicting the outcome, and detecting nonlinear associations. These approaches enable the development of models that more accurately represent the relationship between multiple predictors and the outcome in a regression setting [20]. This data-driven method allows the effect of each predictor on the outcome to be nonlinear and estimated from the data. In the case of multi-predictors, some predictors might have a linear effect and others nonlinear. In this situation, the GAM can be a mixture of linear and nonlinear effects, especially if there is a categorical predictor in the model. Not only the main effects, but also the interaction of two or more predictors, can be assumed as the nonlinear terms in the model. Therefore, GAM provides a flexible methodology to analyze a wide range of different types of data sets. The GAM can be formulated by replacing the standard linear model combination of predictors in Equation (1), such as $\sum \beta_{j}X_{j}$ with $\sum f_{j}\left(X_{j}\right)$, where $f_{j}$ is a smooth non-linear function of jth predictor $X_{j}$, respectively, which is estimated from the data [21]. The GAM is written as follows:

(3) $$g\left(E\left(Y\right)\right)=\alpha +f_{1}\left(X_{1}\right)+f_{2}\left(X_{2}\right)+\;\ldots \;+f_{p}\left(X_{p}\right)$$

The function $f_{j}$ in Equation (3) can be estimated in different ways. The basic step of all these methods is a scatter plot smoother. In this approach, a scatter plot transforms into a smooth fitted function. Some of these methods are spline methods, Locally Estimated Scatterplot Smoothing (LOESS), and a linear smoother, such as standard local linear smoother [21]. The estimated function ${\hat{f}}_{j}$ can reveal the nonlinear effect of the predictor $X_{j}$ on the outcome. Boroumand et al. [22] introduced a tilted linear smoother to detect the nonlinear relationship between one predictor and an outcome. They have shown that the performance of a linear smoother can be improved by applying the tilted technique. Tilting is a technique for modifying an empirical distribution, in which 1/n as data weights (where *n* denotes the sample size) are replaced with $p_{i}$ (general multinomial distribution). It is shown that the tilted estimators are consistent and have optimal convergence rates. It means that by using this estimator for estimating $f_{j}\left(X_{j}\right)$, we will have even more accurate results.

In this paper, we propose a novel approach for investigating the association between clinical characteristics of predictors and AOV as the outcome. This novel approach is the Tilted Additive Model (TAM). We use a tilted nonparametric regression estimator to estimate the nonlinear effect of predictors on the outcome in the additive setting for the first time. In other words, we extend the tilted linear smoother method as a novel approach for a multi-predictor setting. We will apply TAM for estimating the $f_{j}$ in the GAM approach, which extends GAM for analyzing AOV in the ocular surface of diabetic patients.

## 2. Materials and Methods

### 2.1. Study Population

There were 334 diabetic patients aged 30 to 60 in this cross-sectional study. They presented to outpatient clinics at the Khatam-Al-Anbia hospital, Mashhad, Iran, from March 2009 to March 2011. The exclusion criteria include any history of severe blepharitis, large pterygium, conjunctivitis, episcleritis, scleritis, uveitis, any condition causing red-eye, or extra-capsular cataract extraction or scleral buckling. Patients who had used contact lenses were also excluded. Mild lid crusting was permitted. Patients with any history of the following diseases were eliminated: dysthyroidism, anemia, allergy, abnormal fasting blood sugar (FBS), or rheumatic disorder. Patients with hypertension were also eliminated. A complete medical history was taken. Then, height, weight, Hypertension Duration (HTN-Dur), and blood pressure were measured. A panel of laboratory tests was done, including FBS measurement, Hemoglobin A1C, Blood Urea Nitrogen (BUN), Serum Creatinine (S-cr), Urine protein (U-pr), Urine Creatinine (U-cr), daily Urinary Protein Excretion (UPE), and Glomerular Filtration Rate (GFR). Conjunctiva photography was then afterward, followed by a complete ophthalmic examination, including funduscopy with dilated pupils.

### 2.2. Photography

**Figure 1:**
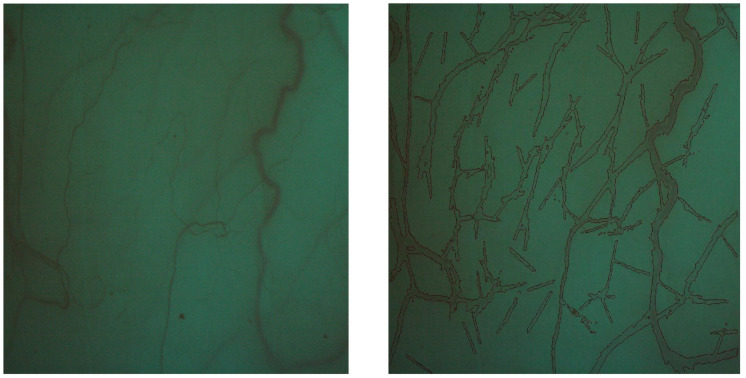
An example of the conjunctival images; the right image shows how the algorithm extracts the vessels.

A YZ5S digital slit lamp microscope (Suzhou 66 Vision-Tech Co., Ltd., Suzhou, Jiangsu, China) was used to take digital red-free conjunctival images of the superior conjunctiva. Photography camera settings were ambient light conditions, a five-volt slit-lamp input, red-free filter, diffuser illumination arm at 45 degrees to the microscope, diffuse illumination (8 mm circle), and 25× magnification. Photography was performed as quickly as possible to minimize dryness and irritation. As the camera is slit lamp mounted, the image is taken at a standard distance, which is the focal point of the slit lamp microscope, and is a fixed distance. In addition, the magnification of the slit lamp and the camera lens were fixed. So the conditions for photography of the eyes was the same with no changes in the magnification of the images. Although we reached a conversion factor of 18 microns/pixel of images, as AOV used in the analyses is a percentage, the unit of output of the algorithm did not change. The algorithm presented in [23] was used for vessel extraction in the conjunctival images. However, this algorithm cannot distinguish between vessels with 2n-and 2n + 1-pixel widths. Thus, the algorithm was modified using Maurer’s distance transform [24]. In this transformation, the Euclidean distance of vessel pixels from backgrounds is estimated in the vessel map image. Analysing these distances provides vessel diameters. The accuracy of the algorithm was studied using several tests on synthetic images, consisting of lines of different diameters in various directions. The estimated accuracy for the algorithm was 98.5%. Finally, the outcome (AOV) was measured. An example of images is provided in Figure 1. The AOV represents a ratio of the image pixels occupied by vessels of different diameters to the total pixels in the picture. More details about the data gathering process are available in [12]. We took the AOV as the outcome variable and evaluated the associations among the predictors and the outcome using the TAM.

### 2.3. Statistical Method

Since the outcome (AOV) is a continous variable, the link function would be the identity so the model in Equation (3) will be

(4) $$Y=\alpha +f_{1}\left(X_{1}\right)+f_{2}\left(X_{2}\right)+\;\ldots \;+f_{p}\left(X_{p}\right)+\epsilon .$$

For fitting the TAM to the data, we need to estimate $f_{j}$ using a tilted linear smoother from [22]. For a single predictor *X*, and *Y* the outcome,

Y=f(X)+ϵ,

*f* can be estimated using the tilted linear smoother

(5) $$\hat{f}\left(x|p,h\right)=\sum_{i=1}^{n}p_{i}l_{i}\left(x_{i}\right)Y_{i},$$

where $p_{i}s$ are tilting parameters and each $p_{i}\geq 0$ and $\sum_{i}^{n}p_{i}=1$, and $l_{i}\left(x\right)$ is the standard local linear, defined as

(6) $$l_{i}\left(x\right)=\frac{b_{i}\left(x\right)}{\sum_{j=1}^{n}b_{j}\left(x\right)},$$

$$b_{i}\left(x\right)=K\left(\frac{X_{i}-x}{h}\right)\left(S_{n}{,}_{2}\left(x\right)-\left(X_{i}-x\right)S_{n}{,}_{1}\left(x\right)\right),$$

$$S_{n}{,}_{j}\left(x\right)=\sum_{i=1}^{n}K\left(\frac{X_{i}-x}{h}\right){\left(X_{i}-x\right)}^{j},$$

j=1,2.

The kernel function *K*, is a weighting function that assigns weights to the observations based on their distance to the target point $x_{0}$. The kernel relies on a parameter named bandwidth outlined by *h*. This parameter determines the maximum distance from the target point to any observation receiving weight. In fact, *h* plays a trade-off role between the bias and variance of estimate. So choosing an optimal *h* plays the main role in the estimation procedure  [25]. By applying the tilted linear smoother method, the optimal *h* and tilting parameters will be estimated to get the optimal convergence rate, which leads to the best estimate in terms of Mean Square Errors (MSE). Using this procedure results in a smooth estimate of *f*, which reveals any nonlinearity effect of *X* on the outcome. With multiple predictors, where $X_{ij}$ denotes the value of the $j^{th}$ predictor for $i^{th}$ observation, the additive model is

(7) $$Y_{i}=\sum_{j}f_{j}\left(X_{ij}\right)+\epsilon_{i}.$$

Here, we assume that the intercept has been absorbed into one of the functions. We use the same approach to estimate each $f_{j}$ during an iterative procedure. The algorithm continues until the estimates do not change from the previous iteration.

$$\begin{array}{cc} f_{1}\left(X_{1}\right) & =Y-•-f_{2}\left(X_{2}\right)-\cdots -f_{p}\left(X_{p}\right)\\ f_{2}\left(X_{2}\right) & =Y-f_{1}\left(X_{1}\right)-•-\cdots -f_{p}\left(X_{p}\right)\\ \vdots \\ f_{p}\left(X_{p}\right) & =Y-f_{1}\left(X_{1}\right)-f_{2}\left(X_{2}\right)-\cdots -• \end{array}$$

Applying this procedure leads to prediction of the outcome in terms of nonlinear function of each predictor. The outcome is AOV and the list of predictors is Age, Gender, BMI, HTN-Dur, daily UPE, GFR, A1C, BUN, Mean Arterial Pressure (MAP), S-cr and Area. The predictor Area with four categories refers to different parts of the eyes, 1 right eye top, 2 right eye bottom, 3 left eye top, and 4 left eye bottom. The degrees of freedom (df) for the linear model equals *p* (p−1 is the number of predictors). It also can be written as the rank of the design matrix. The df for the TAM is the trace of the smoother matrix for each term. It could be said that the df for each term in the additive setting shows the complexity of each estimated function. If we have two fitted responses, a linear model and a tilted local linear smoother, we can compare two models by comparing the decrease in the Residual Sum of Squares (RSS) due to fitting a more complex smooth with the increase in df [26]. In the first step, we checked the linearity vs. non-linearity effect of each predictor by fitting GLM and TAM and decide based on any change in RSS. The linearity vs. nonlinearity of each predictor was tested using F test in the presence of all the predictors. We also used the bootstrap technique to provide a confidence band for curve estimates. We did all the programming in R (version 4.0.0., R Foundation for Statistical Computing, Vienna, Austria). The glm package was used for fitting the GLM, and we performed programming for fitting the TAM, the proposed method, and confidence band of the estimated curve. The code is available in Appendix A.

## 3. Results

Because some of the photographs for the 334 diabetic patients in the study were blurry, the number of images used for analysis was reduced to 297. Eventually, we reached 168 (60 patients were female) complete individual (without missing) records for assessing the associations among predictors and the outcome. The demographic and clinical characteristics of 168 diabetic patients are presented in Table 1. The vessel diameters from 4–53 pixels (72–954 μ m) were detected using the algorithm explained in [12], and AOV was obtained.

**Figure 2:**
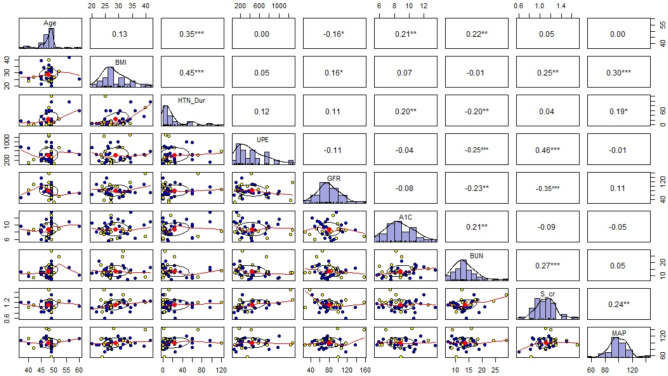
A scatter plot matrix shows the relationship between each pair of predictors. In the diagonal direction, the distribution of each predictor is provided. The upper end of the diagonal shows the Pearson correlation between each pair of predictors. Significant codes: ‘***’ 0.001, ‘**’ 0.01,‘*’ 0.05. BMI = Body Mass Index; HTN-Dur = Hypertension Duration; UPE = daily Urinary Protein Excretion; GFR = Glomerular Filtration Rate; BUN = Blood Urea Nitrogen; S-cr = Serum Creatinine; MAP = Mean Arterial Pressure

Figure 2 shows the relationship between each pair of predictors. It also provides the distribution of each predictor along the diagonal. The Pearson correlation between each pair of predictors is provided in the upper diagonal. The significant correlations are highlighted. The correlation ellipses are also provided in Figure 2. The narrower ellipse shows the greater correlation between the predictors. The wider and more round the ellipse, the more the predictors are uncorrelated.

It can be seen from Figure 2 that all the predictors are distributed approximately symmetrically, except for daily UPE and HTN-Dur, which are right-skewed. The distribution of predictors does not significantly deviate from Normal distribution. Thus, the Pearson correlation coefficient can be estimated. The Pearson correlation between Age and HTN-Dur, GFR, Hemoglobin A1C, and BUN is significant. The positive correlation between Age and HTN-Dur (ρ=0.22) is as expected. There is a negative correlation between Age and GFR (ρ=−0.16) and a positive correlation between Age and Hemoglobin A1C (ρ=0.21). BMI has a positive significant correlation with HTN-Dur (ρ=0.45), and with GFR (ρ=0.16) and with S-cr (ρ=0.25) and with MAP (ρ=0.30). For Diabetic patients, there is a significant positive correlation between HTN-Dur and Hemoglobin A1C (ρ=0.20) and between HTN-Dur and MAP (ρ=0.19). There is also a significant negative correlation between HTN-Dur and BUN (ρ=−0.20). Daily UPE has a negative significant correlation with BUN (ρ=−0.20) and a positive significant correlation with S-cr (ρ=0.46). It is also notable that GFR has a significant negative correlation with BUN (ρ=−0.23) and S-cr (ρ=−0.35). There is a significant positive correlation between Hemoglobin A1C and BUN (ρ=−0.23), BUN and S-cr (ρ=0.17) and S-cr and MAP (ρ=0.24).

Under the diagonal in Figure 2, scatter plots of each pair of predictors are provided. Yellow points refer to female diabetic patients, and blue points refer to male diabetic patients. Since the blue and yellow points are scattered approximately the same, we can conclude that the relationship between each pair of predictors for male and female patients is the same.

**Figure 3:**
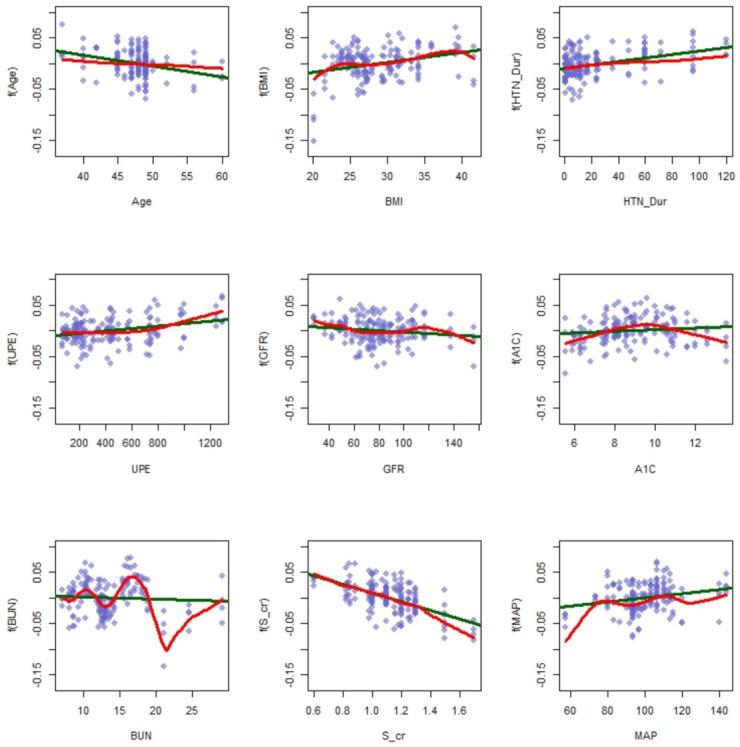
The red curves in the nine panels of this figure are the estimated effects f(x), obtained by fitting Y=f(x) where *x* is Age, BMI, HTN _ Dur, daily UPE, GFR, A1C, BUN, S-cr, and MAP; the green lines in the panels are the linear fit of every predictor.

Figure 3 provides the linear vs. the nonlinear fit for all the predictors. Comparing both fits (linear vs. nonlinear) reveals that the predictors including Age, HTN-Dur, and S-cr have linear effects, while BMI, daily UPE, GFR, A1C, BUN, and MAP have nonlinear effects on the outcome, AOV. Figure 3 supports that a nonlinear model should be employed. A linear model can not detect any significant effect of these predictors since the linear fit is close to Y=0, while they clearly can predict the outcome in the nonlinear term. The previous research on this data set could not detect significant effects of these predictors on the outcome, using a linear model. More details are available in [12].

To support the results presented in Figure 3, the F test of the linearity vs. nonlinearity of each predictor in the presence of other predictors is presented in Table 2. This test shows whether the decrease in RSS by adding the nonlinear effect is significant or otherwise. We can see from the P-value in the last column of Table 2 that Age, BMI, daily UPE, Hemoglobin A1C, and BUN have a nonlinear effect on AOV, while the F test suggests adding HTN-Dur, GFR, S-cr, and MAP to the model as linear effects. Therefore, we modified the final model by changing the effect of HTN-Dur, GFR, S-cr, and MAP from nonlinear to linear. It is worth noting that Figure 3 suggests adding Age as a linear term to the model. However, it is not confirmed by the F test (*p*-value = 0.0437). Since the *p*-value is on the borderline, we decided to add it as a linear term. It is always preferable to fit a simple model instead of a complex one.

**Figure 4:**
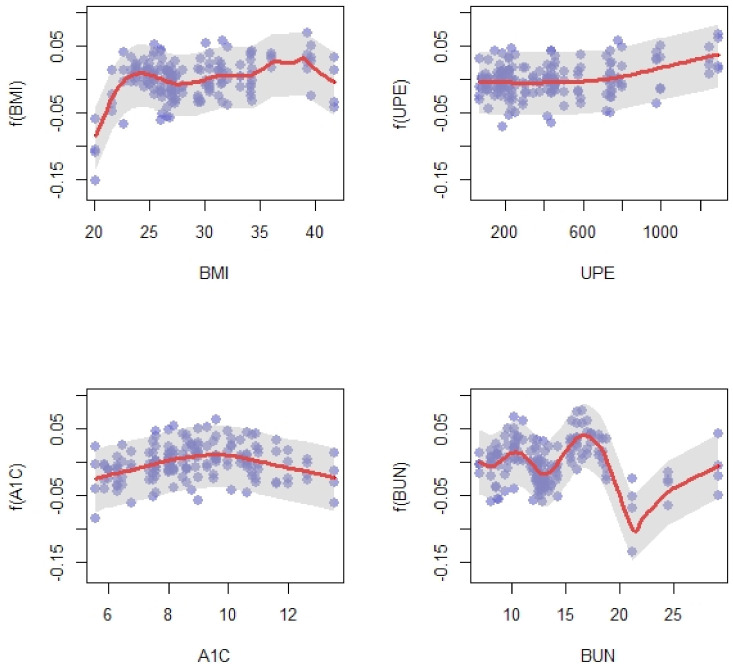
The red curves are the fitted functions for the model: $Y=f_{BMI}\left(BMI\right)+f_{UPE}\left(UPE\right)+f_{A1C}\left(A1C\right)+f_{BUN}\left(BUN\right)+Age*\beta_{Age}+HTN-Dur*\beta_{HTN-Dur}+GFR*\beta_{GFR}+S-cr*\beta_{S-cr}+MAP*\beta_{MAP}$ using the Tilted Additive Model (TAM) approach to estimate fj. The shaded areas represent point-wise nonparametric bootstrap confidence intervals.

The final model is a combination of parametric (linear) and nonparametric (nonlinear) terms. It means that the final model is a semiparametric model. The results of the final model are provided in Figure 4, which shows the nonlinear effects, and Table 3, which provides the linear effects.

The nonlinear trend is apparent. For the following interpretations, it is assumed that other predictors are fixed. The association between AOV and BMI is presented in the top-left panel of Figure 4. As you can see, the AOV has an upward trend as the BMI increases; the first maximum for BMI is around 24 and then it fluctuated gradually before reaching the second maximum for BMI of 39. The top-middle panel shows the variation of AOV in regards to daily UPE. AOV remained steady for 100 < daily UPE < 700 and then increased slightly. The bottom-right panel shows the variation of AOV vs. hemoglobin A1C. As hemoglobin A1C increased, AOV increased before reaching a peak for hemoglobin A1C around nine then decreasing negligibly. The bottom-left panel refers to BUN, AOV for those who have 5 < BUN < 15 oscillated, and then dropped for 15< BUN <20 before increasing again.

In Table 3, the linear effects are provided. The table shows that Age has a negative significant linear effect on AOV. For every unit increase in Age, AOV decreased 0.002. Another significant predictor is MAP. The results show a positive association between MAP and AOV. For every unit increase in MAP, AOV increased 0.0005. Area, with four categories is defined as follows. One refers to the right eye top. Two refers to the right eye bottom. Three refers to the left eye top, and four refers to the left eye bottom. The results show that AOV in Area three is significantly different from others. We could not find any clinical reason for this finding in the literature. The other predictors, Gender, HTN-Dur, and S-cr do not have a significant effect on AOV. However, we kept them in the model to adjust their probable confounding effect.

**Figure 5:**
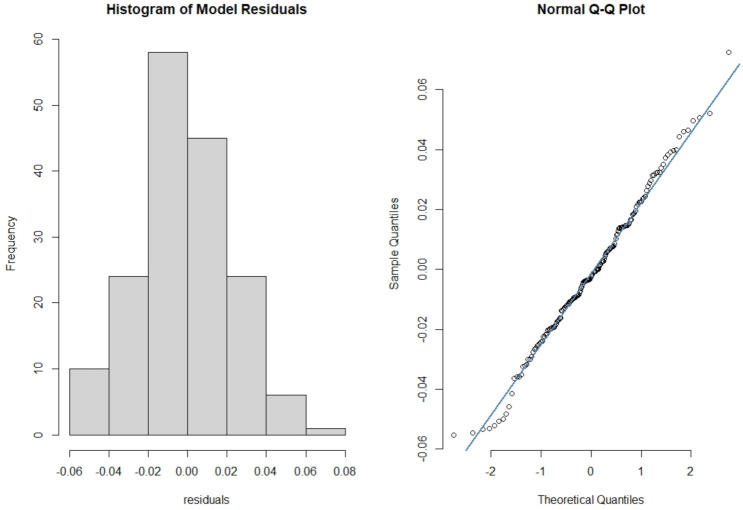
Shapiro-Wilk normality test, W = 0.99288, *p*-value = 0.5819. There is no significant deviation from normal distribution.

In Figure 5, we checked the goodness of fit of the final model by analyzing the deviance residuals. The analysis of deviance residuals reveals that the residuals follow Normality distribution.

## 4. Discussion

In this research, we found a nonlinear association between clinical characteristics of daily UPE, Hemoglobin A1C, and BUN with the AOV in the ocular surface in diabetic patients. The linear associations between Age and MAP and AOV are notable. AOV decreases with increasing age and increases with increasing blood pressure. The model shows that with increasing BMI, the AOV increases, although with some fluctuations. This is consistent with the linear relation between the AOV and MAP as both increased BMI and elevated blood pressure are part of the metabolic syndrome [27] and may have a common pathophysiologic effect on the blood vessel smooth muscles. This also reflects the correlations between BMI and MAP found in Figure 2. Most of the correlations between the predictor in Figure 2 are clinically relevant. AOV increases with an increase in hemoglobin A1C and peaks when the A1C level is 9%. Increasing hemoglobin A1C by more than 9% has the reverse effect, causing lower AOV values. We still do not have an explanation for this relationship, but it seems to lie in the delicate biochemical balance of advanced glycation end-product formation and the function of the vascular smooth muscle cells. The effect of daily UPE on AOV is not discernible until a daily excretion of more than 650 mg/d. Higher amounts of protein excretion are associated with an increase in AOV. Some proteins such as vasopressin and angiotensin-converting enzyme, which have roles in vasoconstriction, can be excreted in urine [28]. We hypothesize that at this level of proteinuria, significant amounts of these proteins may be lost in the urine, hence resulting in vasodilation, although it may be an oversimplistic explanation for such a condition. The relation of BUN with AOV fluctuates but because BUN levels in this study fall mostly in the normal range, we could not find a clinically significant association. There is considerable literature examining the effect of diabetes on retina. However, there are few studies concerning the effect of DM on the ocular surface. We cannot imagine an immediate clinical application for our findings, but they are useful for future studies on the ocular surface microvasculature. We found nonlinear associations of predictors on AOV in diabetic patients that show diabetes may involve the conjunctival and episcleral vessels. Since the conjunctival vessels are more accessible, it offers an opportunity for future research to use conjunctival vessels as a marker of diabetic retinopathy.

The nonlinear modeling procedure developed here—the TAM, an extension of GAM with identity link function—is useful for three reasons. First, using this model prevents model misspecification. Model misspecification can lead to incorrect conclusions. Second, it reveals the nonlinear associations among the predictors and the outcome that cannot be identified using standard modeling techniques. Moreover, a linear association can be detected in this setting as a particular case. Third, it is easy to interpret, which means these types of modeling can be widely used in medical research. Hastie and Tibshirani [19] have provided the most comprehensive source for the GAM, describing generalized additive models as adjustable statistical methods that can be utilized to detect and model the nonlinear effect of potential predictors of disease outcome. Herman and Hastie [29] used nonparametric multiple logistic regression as a form of GAM to examine the relationship between gestational age, neonatal size, and neonatal death. They showed that gestational age has a nonlinear effect on neonatal death. Charytanowicz and Kulczycki [30] used the same approach for analyzing the correlation of hematologic parameters with creatinine for patients with renal insufficiency observed in the Stefan Kardynal Wyszynski Regional Specialists’ Hospital in Lublin (Poland). Seposo et al. [31] conducted a multi-city study carried out in tropical cities, evaluating the temperature–-diabetes relationship. Using GAM for analysing the data in this study revealed a nonlinear association between temperature and the risk of diabetes.

Using a tilting approach to estimate $f_{j}\left(x\right)$ in a GAM setting leads to improvement of the performance of the nonparametric regression estimator. Hall and Presnell [32], Hall and Huang [33], Carroll [34], Doosti and Hall [35], and Doosti et al. [36] used setup-specific Distance Measure approaches for estimating the tilting parameters. Carroll [34] proposed a new approach for density function estimation, and regression function estimation, as well as hypothesis testing under shape constraints in the model with measurement errors. A tilting method used in [34] led to curve estimators under some constraints. Doosti and Hall [35] introduced a new higher order nonparametric density estimator, using a tilting method, where they used $L_{2}$-metric between the proposed estimator and a consistent ’Sinc’ kernel-based estimator. Doosti et al. [36], have introduced a new way of choosing the bandwidth and estimating the tilted parameters based on the cross-validation function. In [36], it was shown that the proposed density function estimator had improved efficiency and was more cost-effective than the conventional kernel-based estimators studied in this paper. Boroumand et al. [22] proposed a new tilted version of a linear smoother nonparametric regression that is obtained by minimizing the distance to a comparator estimator. The comparator estimator is selected to be an infinite-order flat-top kernel estimator. They proved that the tilted estimators achieve a high level of accuracy, yet preserving the attractive properties of an infinite-order flat-top kernel estimator. In this study, we used the tilted linear smoother in an additive setting for the first time as a novel approach to identify nonlinear effects. We were able to detect the nonlinear association among clinical characteristics and AOV in diabetic patients, while previous research on this data set could not identify the significant effects of clinical characteristics on AOV using a linear model [12].

## 5. Conclusions

This study shows that Hemoglobin A1C and daily urinary excretion of protein affect the ocular surface microvasculature. These findings may not have instant clinical utility but will be helpful in research using ocular surface photography for evaluation of systemic factors. A Tilted Additive Model can find correlations that were missed in a previous study on the same data set [12] using a linear regression model. This statistical method is a valuable tool to search for nonlinear relationships, which are common in the biomedical field. We detect significant nonlinear associations using a novel approach. As many predictors do not have a linear relationship with the outcome, the Tilted Additive Model is a novel approach that helps to elucidate the effect of different predictors.

## Figures and Tables

**Table 1:** Demographic and clinical characteristics of 168 diabetic patients.

Predictor	Mean	Standard Deviation
Age	47.79	3.70
Body Mass Index (BMI) (kg/m${}^{2}$)	28.66	4.81
Hypertension Duration (HTN-Dur)	23.98	29.56
daily Urinary Protein Excretion (UPE)	433.3	310.70
Glomerular Filtration Rate (GFR)	80.14	25.76
Hemoglobin A1C	8.824	1.85
Blood Urea Nitrogen (BUN)	13.55	4.44
Fasting Blood Sugar (FBS)(mg/dL)	166.20	54.76
Serum Creatinine (S-cr) (mg/dL)	1.11	0.20
Mean Arterial Pressure (MAP) (mg/dL)	100.06	15.43

**Table 2:** The linear effect vs. nonlinear effect of each predictor in the presence of other predictors; columns (1)–(6) refer to predictors, Residual Some of Squares Linear (RSS L), Residual Some of Squares nonlinear (RSS N), degrees of freedom Nonlinear (df N), *F* value, and *p*-Value; ** significant at 0.001, * significant at 0.05.

Predictor	RSS L	RSS N	df N	*F* Value	*p*-Value
Age	0.091	0.094	3.015	−3.181	0.0437 *
Body Mass Index (BMI)	0.143	0.122	3.880	9.397	<0.001 **
Hypertension Duration (HTN-Dur)	0.091	0.095	4.821	−2.023	0.097
Daily Urinary Protein Excretion (UPE)	0.094	0.091	3.093	3.264	0.038 *
Glomerular Filtration Rate (GFR)	0.096	0.090	8.913	1.293	0.251
Hemoglobin A1C	0.107	0.090	7.759	4.275	<0.001 **
Blood Urea Nitrogen (BUN)	0.181	0.099	8.736	17.085	<0.001 **
Serum Creatinine (S-cr)	0.093	0.093	8.227	−0.146	0.995
Mean Arterial Pressure (MAP)	0.114	0.116	6.770	−0.364	0.895

**Table 3:** The linear part of the final model; ** significant at 0.01, * significant at 0.05.

Predictor	Estimate	Std. Error	*t* Value	*p*-Value
Intercept	0.0654	0.0891	0.733	0.4646
Age	−0.002	0.001	−2.373	0.019 *
Gender (Female/male)	−0.003	0.007	−0.365	0.716
Hypertension Duration (HTN-Dur)	0.0002	0.0001	1.436	0.153
Glomerular Filtration Rate (GFR)	−1.232 × 10−6	1.791 × 10−4	−0.007	0.994
Serum Creatinine (S-cr)	−0.036	0.0348	−1.036	0.302
Mean Arterial Pressure (MAP)	0.0005	0.0002	2.728	0.007 **
Area (2/1)	−0.0013	0.0057	−0.231	0.818
Area (3/1)	0.0123	0.0057	2.142	0.034 *
Area (4/1)	0.0075	0.0057	1.315	0.191

## Data Availability

Restrictions apply to the availability of these data. Data was obtained from Mashhad University of Medical Sciences, Mashhad, Iran, and are available with the permission of Mashhad University of Medical Sciences.

## Appendix A. R Code for Statistical Analysis

#-----------------------------------------Data and bandwidths
res<-read.csv("")
var<-read.csv("")
h_ll=c(2.845401,1.587784,9.152896,
108.1861,12.0652,0.5424099,0.5,0.2152706,8.7255)
h_kt=c(5.5,1/0.4,50,1/0.01,1/0.15
,1/2,1/0.65,1/10,1/0.11)
#-----------------------------------------Bootstrap function for CI
library(boot)
Qfun <- function(data, i){
  d <- data[i, ]
  return(quantile(d,probs = c(0.025, 0.975)))
}
#-----------------------------------------Calculating df
library(psych)
l_Tllp <- function(x,X,h,p)p*bp(x,h,X)/mean(bp(x,h,X))
l_Tllp=Vectorize(l_Tllp,"x")
#-----------------------------------------TAM fitting
MSE=rep(NA,9)
e=matrix(NA,length(res[,1]),9)
df1=rep(NA,9) #df1=tr(t(S)*S)
df2=rep(NA,9) #df2=tr(S)
df3=rep(NA,9) #df3=tr(2*S-t(S)*S)
micro=c("Age","BMI","HTN_,Dur","UPE","GFR",
"A1C","BUN","S_cr","MAP")
fmicro=c("f(Age)","f(BMI)","f(HTN_Dur)","f(UPE)",
"f(GFR)","f(A1C)","f(BUN)","f(S_cr)","f(MAP)")
par(mfrow=c(3,3))
resid=matrix(NA,length(res[,1]),9)
for (i in 1:9) {
X=var[,i]
Y=res[,i]
n=length(Y)
plot(X,Y,col=rgb(0.4,0.4,0.8,0.6),
pch=16 , cex=1.3 ,ylab=fmicro[i],
xlab =micro[i],ylim=c(-0.17,0.1))
abline(lm(Y~X),col="darkgreen",lwd=3)
resid[,i]=resid(lm(Y~X))
curve(rn_ll(x,Y,X,h=h_ll[i]),min(X),max(X),
add = T,col=2, lwd=3 ,ylim=c(-0.25,0.3))
curve(rn_Iorder(x,h=h_kt[i],X,Y=Y),min(X),max(X)
,add=T,lwd=3,col="blue")
theta_opt <- as.double(try(constrOptim(c(rep(1/n,k-1),h_ll[i]),
targetfunc,gr=NULL,ui=ui,ci=ci,Y=Y,X=X,$par,silent=T))
pk <- k/n-sum(theta_opt[-k])
p <- rep(c(theta_opt[-k],pk),each=n/k)
curve(rn_Tllp(x,Y,X,theta_opt[k],p),min(X),max(X),
add=T,lwd=3,col="red",ylab=fmicro[i], xlab =micro[i])
#rug(X, ticksize = 0.06, side = 1, lwd =1)
df2[i]=tr(l_Tllp(X,X,h=theta_opt[k],p=p))
e[,i]=Y-rn_Tllp(X,Y,X,theta_opt[k],p)
MSE[i]=mean((e[,i])^2)
eb=(e[,i]-mean(e[,i]))/sd(e[,i]) #standardized e for bootstrapping
data <- data.frame(eb)
bo <- boot(data[,"eb", drop = FALSE], statistic=Qfun, R=5000)
lcb= rn_Tllp(X,Y,X,theta_opt[k],p)+mean(bo$t[,1])*sqrt(MSE[i])
ucb= rn_Tllp(X,Y,X,theta_opt[k],p)+mean(bo$t[,2])*sqrt(MSE[i])
myPredict <- cbind(rn_Tllp(X,Y,X,theta_opt[k],p),lcb,ucb)
ix <- sort(X,index.return=T)$ix
polygon(c(rev(X[ix]), X[ix]), c(rev(myPredict[ ix,3]),
myPredict[ ix,2]), col = rgb(0.7,0.7,0.7,0.4) , border = NA)
}
#---------------------------------Ftest linearity vs. nonlinearity
e2=e^2
dev <- apply(e2, 2, sum)
resid2=resid^2
devlm<- apply(resid2, 2, sum)
fstat=rep(NA,9)
pvalue=rep(NA,9)
for (i in 1:9) {
  fstat[i]=((devlm[i]-dev[i])*(168-df2[i]))/((dev[i])*(df2[i]-1))
  pvalue[i]=1-pf(abs(fstat[i]),df2[i]-1,168-df2[i])
}
RSSlm=devlm
RSS=dev
table=cbind(micro,RSSlm,RSS,df2,fstat,pvalue)
#-----------------------------------------Residuals normality plot
par(mfrow=c(1,2))
hist(e[,5], xlab="residuals",main= "Histogram⊔of⊔Model⊔Residuals")
qqnorm(e[,5], pch = 1,frame = FALSE)
qqline(e[,5], col = "steelblue", lwd = 2)
shapiro.test(e[,5])
